# Proteomic analysis of blood serum in bipolar disorder

**DOI:** 10.1192/j.eurpsy.2024.893

**Published:** 2024-08-27

**Authors:** L. Smirnova, A. Seregin, E. Dmitrieva, M. Zavialova, S. Ivanova

**Affiliations:** ^1^Laboratory of Molecular Genetics and Biochemistry, Mental Health Research Institute, Tomsk National Research Medical Center, Russian Academy of Sciences, Tomsk; ^2^Advanced Mass Spectrometry Core Facility, Skolkovo Institute of Science and Technology, Moscow, Russian Federation

## Abstract

**Introduction:**

Bipolar disorder (BD) often has symptoms similar to other mental disorders (BD), and there are no paraclinical criteria for differential diagnosis. (Geoffroy *et al.* Bip Dis 2017; 5 7). Published work on MD proteomics is scarce and focused on schizophrenia. (Dmitrieva *et al.* PeerJ. 2022; 10 e13907). Therefore, it is important to study potential biomarkers of BD using easily accessible material—blood serum (Rhee *et al. Transl Psy* 2023; 13 44). Identification of proteins involved in the pathogenesis of BD will help in the study of the pathogenetic mechanisms of BD, the development of differential diagnostic methods and pathogenetically based drugs.

**Objectives:**

Carrying out a comparative proteomic analysis of blood serum from patients with BD and healthy individuals to identify potential biomarkers

**Methods:**

We analyzed the protein spectrum of the blood serum of 14 patients with BD who were admitted during a depressive episode at the age of 32 [21;52] years with a disease duration of 8[5;11] years. The control group consisted of 10 mentally and somatically healthy individuals corresponding to the gender and age of the BD group. Blood serum was purified from 14 major proteins using affinity chromatography and separated by electrophoresis using the Laemmli method. After trypsinolysis, proteins were identified using HPLC/mass spectrometry on an Orbitrap instrument. Mass spectrometric analysis was performed on the Advanced Mass Spectrometry Core Facility of Skolkovo Institute of Science and Technology. Protein identification was carried out using the UniProtKB database using the Mascot search engine. The results were tested for significance using the nonparametric Fisher exact test with Yates correction.

**Results:**

In patients with BD, qualitative mass spectrometry revealed differential expression of 21 neurospecific proteins. Among them: Protein dispatched homolog 3, Ceroid-lipofuscinosis neuronal protein 6, SWI/SNF complex subunit SMARCC1, Neurogenic differentiation factor 4, Protein furry homolog-like, REST corepressor 1 – are involved in the proliferation, development and differentiation of neurons; Hemicentin-2, Dystrophin, Voltage-dependent L-type calcium channel subunit alpha-1D, Syntaxin-binding protein 5, Small conductance calcium-activated potassium channel protein 1– participate in synaptic transmission of ion transport and form receptors.

**Conclusions:**

Studying the role of these proteins in BD and their quantitative content in a larger number of patients is promising. This will help in the development of new diagnostic criteria and targets for drug therapy for BD.

Support by the Russian Science Foundation grant No. 23-75-00023.

**Disclosure of Interest:**

None Declared

